# GCN2 contributes to mTORC1 inhibition by leucine deprivation through an ATF4 independent mechanism

**DOI:** 10.1038/srep27698

**Published:** 2016-06-14

**Authors:** Julien Averous, Sarah Lambert-Langlais, Florent Mesclon, Valérie Carraro, Laurent Parry, Céline Jousse, Alain Bruhat, Anne-Catherine Maurin, Philippe Pierre, Christopher G. Proud, Pierre Fafournoux

**Affiliations:** 1 INRA, UMR 1019 Nutrition Humaine, Centre de Clermont-Ferrand-Theix, F-63122 Saint Genès Champanelle, France; 2 Université Clermont 1, UFR Médecine, UMR 1019 Nutrition Humaine, Clermont-Ferrand, France; 3 Centre d’Immunologie de Marseille-Luminy, Aix-Marseille Université, U2M, 13288 Marseille, France; 4 INSERM, U1104, 13288 Marseille, France; 5 CNRS, UMR 7280, 13288 Marseille, France; 6 Institute for Research in Biomedicine - iBiMED and Aveiro Health Sciences Program, University of Aveiro, 3810-193 Aveiro, Portugal; 7 Nutrition and Metabolism, South Australian Health & Medical Research Institute, PO Box 11060, Adelaide, SA5001, Australia; 8 School of Biological Sciences, University of Adelaide, Australia

## Abstract

It is well known that the GCN2 and mTORC1 signaling pathways are regulated by amino acids and share common functions, in particular the control of translation. The regulation of GCN2 activity by amino acid availability relies on the capacity of GCN2 to sense the increased levels of uncharged tRNAs upon amino acid scarcity. In contrast, despite recent progress in the understanding of the regulation of mTORC1 by amino acids, key aspects of this process remain unsolved. In particular, while leucine is well known to be a potent regulator of mTORC1, the mechanisms by which this amino acid is sensed and control mTORC1 activity are not well defined. Our data establish that GCN2 is involved in the inhibition of mTORC1 upon leucine or arginine deprivation. However, the activation of GCN2 alone is not sufficient to inhibit mTORC1 activity, indicating that leucine and arginine exert regulation via additional mechanisms. While the mechanism by which GCN2 contributes to the initial step of mTORC1 inhibition involves the phosphorylation of eIF2α, we show that it is independent of the downstream transcription factor ATF4. These data point to a novel role for GCN2 and phosphorylation of eIF2α in the control of mTORC1 by certain amino acids.

The control of cell growth is tightly coupled to the level of protein synthesis, which in turn is controlled by nutrient availability. Among nutrients, amino acids exert a major role on protein synthesis regulation through the control of the kinases mTOR (mammalian [or mechanistic] Target Of Rapamycin) and GCN2 (General Control Non-derepressible 2) which have opposing effects on protein synthesis. In mammals, the potential regulatory interactions between these two pathways are still poorly understood. mTOR, as part of the complex mTORC1, is activated by the presence of amino acids^1^ and phosphorylates the eIF4E-binding proteins, the S6 kinases and eukaryotic elongation factor 2 kinase, all of which events favor protein synthesis^2^,^3^. GCN2 is activated during scarcity of an essential amino acid and phosphorylates the α-subunit of eukaryotic initiation factor 2 alpha (eIF2α)^4^. This leads to the general inhibition of protein synthesis. However, the phosphorylation of eIF2α actually promotes the translation of certain mRNAs that possess uORFs (upstream Open Reading Frames) in their 5′-UTRs, an example of such an mRNA being that encoding the transcription factor activating transcription factor 4 (ATF4)^5^. ATF4 plays a key role in the adaptation of the cell to the lack of amino acids by regulating the expression, of amino acid transporters^6^, enzymes involved in amino acid metabolism^7^ or factors involved in autophagy^8^.

Recent years have seen important advances in the understanding of the regulation of mTORC1 by amino acids^9^. The current model involves the control of mTOR localization at the level of the lysosomal membrane by the Rags, a family of small GTPases^10^,^11^. However, the lysosome is not the only location where mTORC1 can be activated^12^,^13^. This illustrates that our understanding of the mechanisms involved in the sensing of amino acids remains incomplete. There is another level of complexity in the regulation of mTORC1 by amino acids regarding the effects of a starvation/stimulation of all amino acids or for a single amino acid. In particular, leucine has been the focus of many studies, as it is one of the most potent regulators of mTORC1 signaling^14^. The mechanisms involved in the regulation of mTORC1 by leucine or by other amino acids seem to be distinct; in particular, leucine can control mTORC1 activity without modifying the lysosomal localization of mTOR^12^. In addition, it is evident that the mechanisms involved in the sensing of all the amino acids or leucine alone are, at least in part, distinct.

Beside its action on mTORC1 signaling, it is well established that leucine is a potent regulator of the kinase GCN2. The activation of GCN2 by amino acid starvation is mediated by the levels of uncharged tRNA; such tRNAs bind to and activate GCN2^15^. Previous work has established that GCN2 participates in the regulation of mTORC1 activity by leucine^16^,^17^. These two studies observed that a diet devoid of leucine failed to inhibit mTORC1 activity in the liver of GCN2^−/−^ mice, whereas it did impair mTORC1 signaling in wild-type mice. This suggests that GCN2 acts upstream of mTORC1. In agreement with this hypothesis, an analogous result was observed in HepG2 cells knocked-down for GCN2 and starved for leucine for 24 hours^16^. It is conceivable that a transcriptional target of ATF4 mediates the inhibitory effect of GCN2 activation on mTORC1. Indeed, in contexts other than amino acid deprivation, such as endoplasmic reticulum stress or virus infection, two targets of ATF4, REDD1 and GADD34, are known to inhibit mTORC1 activity^18^,^19^. However, since the inhibition of mTORC1 signaling by leucine deprivation is rapid^12^, the contribution of such transcriptional mechanisms is questionable given the inherent delay between increased translation of ATF4, the rise in ATF4 protein levels and subsequent downstream transcription-dependent events.

This study aimed at investigating more deeply the contribution of GCN2 to the regulation of mTORC1 by leucine deprivation. Our data demonstrate that GCN2 is necessary for the early effect of leucine deprivation on mTORC1 signaling and that this operates independently of ongoing transcription. Furthermore, our data establish that the phosphorylation of eIF2α is necessary but not sufficient for the inhibition of mTORC1 during leucine deprivation. Importantly, we show that the role of GCN2 in the inhibition of mTORC1 is not restricted to leucine deprivation, since the ability of arginine deprivation to inhibit mTORC1 also depends on GCN2.

## Results

### GCN2 is necessary for mTORC1 inhibition by leucine deprivation

**Figure 1:**
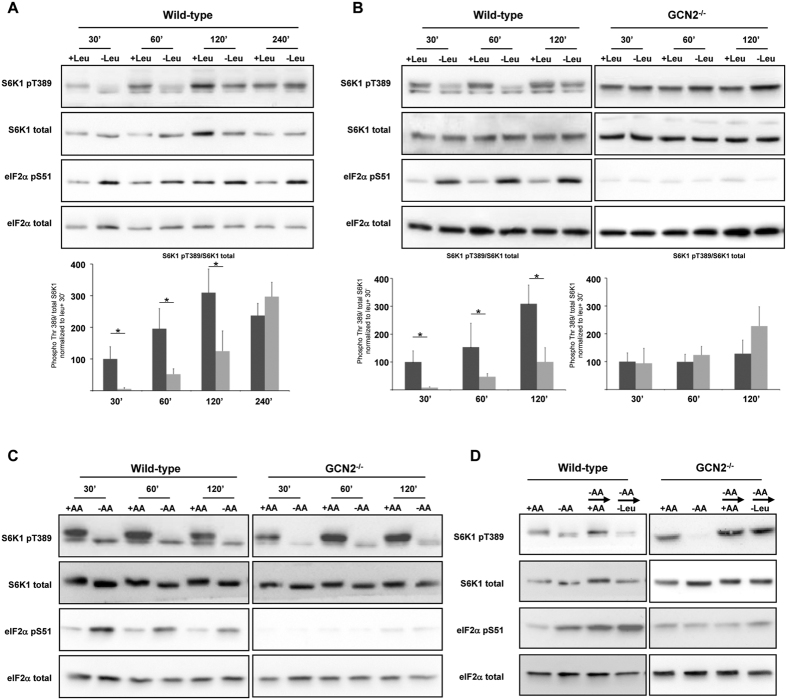
GCN2 is necessary for mTORC1 regulation by leucine. (**A**) Wild-Type (WT) MEFs were transferred to fresh medium containing dialysed serum and all amino acids except leucine, and then maintained in the presence or in absence of leucine (Leu) for the times indicated (minutes). Immunoblot analyses were performed on the resulting cell lysates using the indicated antibodies. The ratio of phosphorylated S6K1 (Thr389) to total S6K1 was determined by densitometry analysis, differences between control cells (dark bars)and leucine starved cells (light bars) at each time point were assessed by 1-way ANOVA. Bars with (*) are significantly different from each other (*P* < 0.05). (**B**) WT or GCN2-KO (−/−) MEFs were maintained in all amino acids including or excluding leucine (Leu) for the times indicated. The ratio of phosphorylated S6K1 (Thr389) to total S6K1 was determined by densitometry analysis, differences between control cells and leucine starved cells at each time point were assessed by 1-way ANOVA. Bars with (*) are significantly different from each other (*P* < 0.05). (**C**) WT or GCN2^−/−^ MEFs were cultured in presence or in absence of all amino acids (AA) for the times shown. (**D**) WT and GCN2^−/−^ MEFs were kept in the presence (+AA) or absence (−AA) of AA for 75 min; or in the absence of AA for 60 min prior to addition for 15 min of a medium containing all AA (−AA → + AA) or a medium containing all AA except leucine (−AA → -Leu). In all panels, immunoblot analyses were performed on the resulting cell lysates using the indicated antibodies.

To examine the role of GCN2 in the inhibition of mTORC1 activity we first performed a time-course of leucine deprivation in mouse embryonic fibroblasts (MEFs). By 30 min, leucine starvation increased the phosphorylation of eIF2α (Fig. 1A) and also inhibited mTORC1 activity as shown by the decrease in the level of phosphorylation of its direct substrate S6 kinase 1 (S6K1). The effect of leucine deprivation on S6K1 phosphorylation was transient, a recovery of the phosphorylation being observed between 2 and 4 h of starvation. This phenomenon may reflect the fact that the induction of proteolysis, and notably autophagy, upon leucine deprivation may enhance the intracellular levels of the missing amino acid^8^,^20^.

We then focused on the shorter times of starvation and explored the effect of the absence of the eIF2α kinase GCN2 on the regulation of S6K1 phosphorylation. As expected, leucine deprivation failed to induce eIF2α phosphorylation in GCN2^−/−^ MEFs (Fig. 1B). In contrast to the situation for the wild-type (WT) MEFs, we did not observe a consistent or significant decrease in S6K1 phosphorylation during leucine deprivation, at any time-point tested. This observation was confirmed by the study of the phosphorylation of 4E-BP1, the second well-known target of mTORC1 (Fig. S1). In the WT MEFs a decrease of the phosphorylation was observed after 30 and 60 minutes of leucine deprivation whereas no effect was observed in the GCN2^−/−^ MEFs. The level of mTOR phosphorylation at serine 2448 was also assessed. A moderate but significant decrease of the phosphorylation of this site was measured in the WT MEFs after 30 minutes of leucine deprivation. Consistently with the level of phosphorylation of S6K1 and 4E-BP1, no decrease of mTOR phosphorylation was observed in the GCN2^−/−^ MEFs following leucine deprivation. This result implies that GCN2 is necessary for the rapid inhibition of mTORC1 activity by leucine deprivation.

Since the mechanisms involved in the regulation of mTORC1 by leucine or by all amino acid appear to differ, we wanted to evaluate the role of GCN2 in the inhibition of mTORC1 during starvation of cells for all amino acids. The data in Fig. 1C show that this manipulation was still able to strongly decrease S6K1 phosphorylation in GCN2^−/−^ MEFs. This demonstrates that GCN2 is not required for the inhibitory effect of general amino acid starvation on mTORC1 signaling, in contrast to the situation of starvation for leucine alone. In order to assess the idea that GCN2 contributes to the rapid modulation of mTORC1 activity specifically by leucine, we took advantage of the ability of total amino acid deprivation to inhibit S6K1 phosphorylation in GCN2^−/−^ MEFs and tested the role of GCN2 in the re-activation of mTORC1 when amino acids are resupplied. Following a one-hour AA starvation (−AA), cells were resupplied for 15 min with all amino acids (+AA) or all amino acids without leucine (-Leu). In WT MEFs, medium lacking leucine was unable to stimulate mTORC1 activity, whereas in GCN2^−/−^ cells this medium did induce S6K1 phosphorylation indicating activation of mTORC1 (Fig. 1D). This result demonstrates that, in the absence of GCN2, leucine is dispensable for the activation of mTORC1 by the other amino acids. Taken together, these experiments demonstrate that GCN2 plays a central role in the regulation of mTORC1 activity by leucine and that this is distinct from the control of mTORC1 signaling by amino acids in general.

### ATF4 is not required for the short-term effect of leucine deprivation on mTORC1 signaling

**Figure 2:**
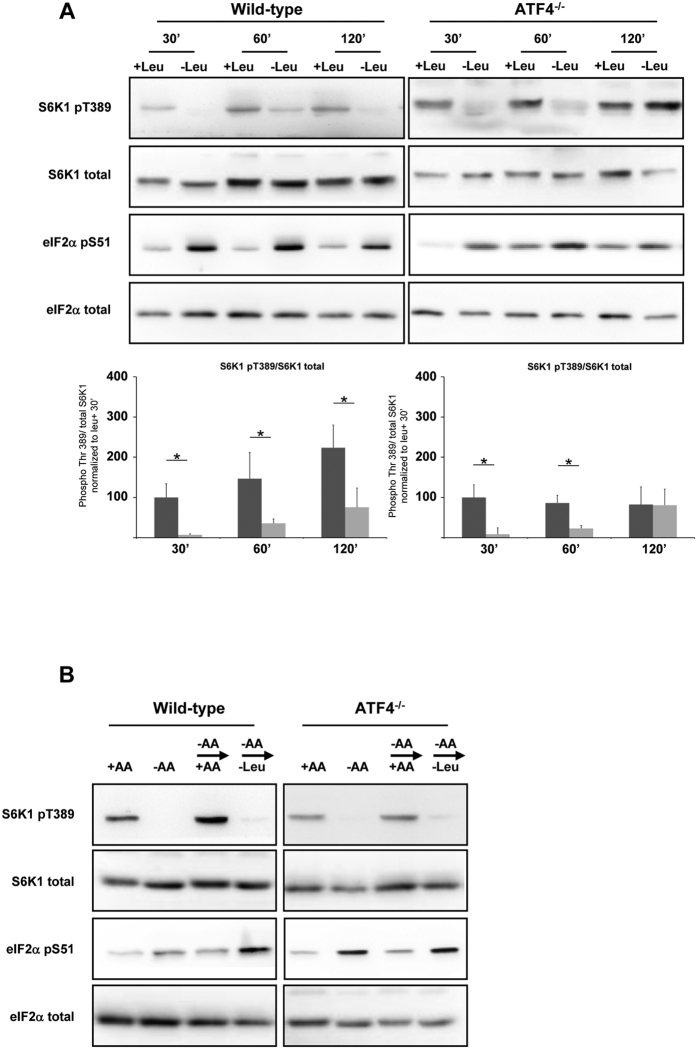
ATF4 is not involved at early times of mTORC1 regulation by leucine. (**A**) WT or GCN2^−/−^ MEFs were cultured in presence or in absence of leucine (Leu) with all the other AAs for 30 min, 60 min or 120 min. The ratio of phosphorylated S6K1 (Thr389) to total S6K1 was determined by densitometry analysis, differences between control cells (dark bars) and leucine starved cells (light bars) at each time point were assessed by 1-way ANOVA. Bars with (*) are significantly different from each other (*P* < 0.05). (**B**) WT MEFs and ATF4^−/−^ MEFs were cultured in the presence or absence of AA for 75 min; or in absence of AA for 60 min prior to addition for 15 min of a medium containing all AA (−AA → + AA) or a medium containing all AA except leucine (−AA → -Leu). In all panels, immunoblot analyses were performed on the resulting cell lysates using the indicated antibodies.

The fact that GCN2 acts in the early events of the regulation of mTORC1 led us to assess the role of ATF4, a transcription factor whose expression is controlled by GCN2^21^, in this process. We investigated the effect of leucine starvation on mTORC1 activity in ATF4^−/−^ MEFs. As in the wild-type cells, inhibition of S6K1 phosphorylation was still seen at the earliest time of leucine starvation (Fig. 2A). The same observation was made for the level of phosphorylation of 4E-BP1 and mTOR (Fig. S1). It can be concluded that ATF4 is not involved in mTORC1 inhibition at early times of leucine starvation. We then performed the same type of experiment of starvation/stimulation as in Fig. 1D. No significant difference was observed between the WT and ATF4^−/−^ cells suggesting that ATF4 is not involved in maintaining mTORC1 inhibition at least in the context of short-term amino acid deprivation (Fig. 2B). Because ATF4 is not the only transcription factor, or protein, that is induced at the translational level by phosphorylation of eIF2α, we also tested the effect of actinomycin D, a transcription inhibitor. This experiment demonstrated that ongoing transcriptional activity is not required for the inhibition of mTORC1 activity by leucine starvation (Fig. S2). These findings emphasize that GCN2 acts independently of ATF4 or any other transcriptional event to regulate mTORC1 signaling.

### The phosphorylation of eIF2α is necessary but not sufficient to inhibit mTORC1 activity upon leucine deprivation

**Figure 3:**
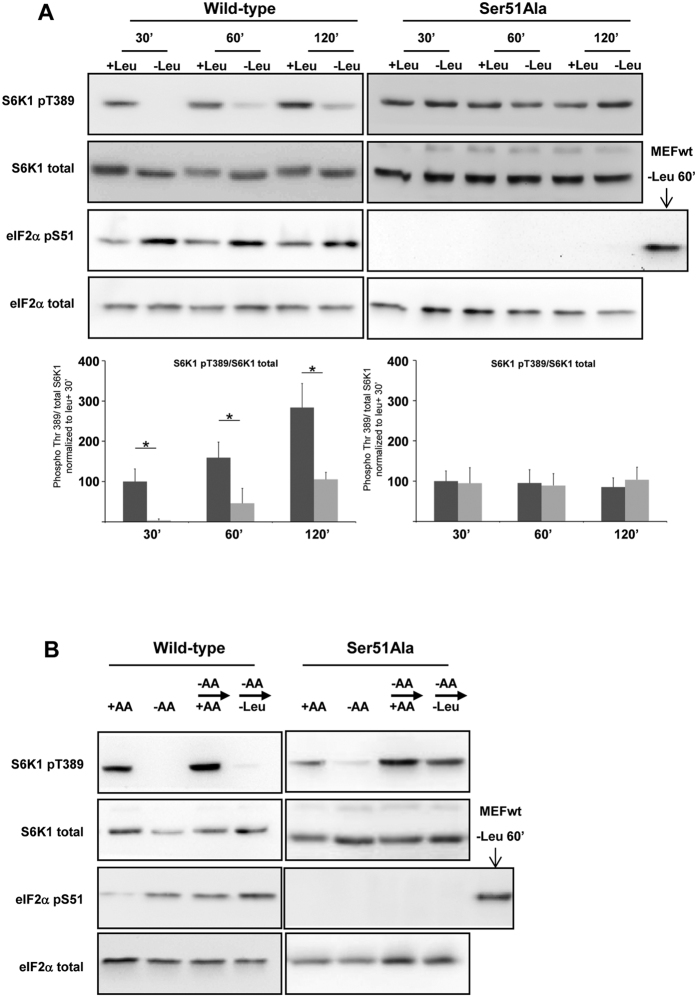
The phosphorylation of eIF2α is necessary for the regulation of mTORC1 by leucine. (**A**) WT or eIF2α[Ser51Ala] MEFs were maintained in presence or in absence of leucine (Leu) with all the other AAs for the times shown. A positive control has been included in the immunoblot analysis for the phosphorylated eIF2α in the eIF2α[Ser51Ala] MEFs. The ratio of phosphorylated S6K1 (Thr389) to total S6K1 was determined by densitometry analysis, differences between control cells (dark bars) and leucine starved cells (light bars) at each time point were assessed by 1-way ANOVA. Bars with (*) are significantly different from each other (*P* < 0.05). (**B**) WT or eIF2α[Ser51Ala] MEFs were kept in the presence or absence of AA for 75 min + AA); or in the absence of AA (−AA) for 60 min prior to addition for 15 min of a medium containing all AA (−AA → + AA) or a medium containing all AA except leucine (−AA → -Leu). A positive control has been included in the immunoblot analysis for the phosphorylated eIF2α in the eIF2α[Ser51Ala] MEFs. Immunoblot analyses were performed to measure the amounts of the indicated proteins and their levels of phosphorylation.

We then examined the role of eIF2α phosphorylation in the regulation of mTORC1 signaling by leucine. For this purpose, we used eIF2α^S51A/S51A^ MEFs in which eIF2α has been mutated at Ser51 and so cannot be phosphorylated. In this cell line, leucine starvation was unable to inhibit S6K1, 4E-BP1 and mTOR phosphorylation (Figs 3A and S1) whereas deprivation of all amino acids did still inhibit S6K1 phosphorylation (Fig. 3B). Furthermore, following the inhibition of mTORC1 activity by the withdrawal of amino acids, addition of medium lacking leucine was still able to induce the phosphorylation of S6K1 (Fig. 3B) in eIF2α^S51A/S51A^ MEFs, whereas this medium was unable to induce S6K1 phosphorylation in WT MEFs. We studied whether leucine deprivation induced activation of GCN2 in eIF2α^S51A/S51A^ MEFs; a significant increase in the phosphorylation of GCN2 at threonine 898 was observed in this cell line (Fig. S3). These results demonstrate that the phosphorylation of eIF2α is a necessary event in the inhibition of mTORC1 by GCN2 activation upon starvation for leucine.

**Figure 4:**
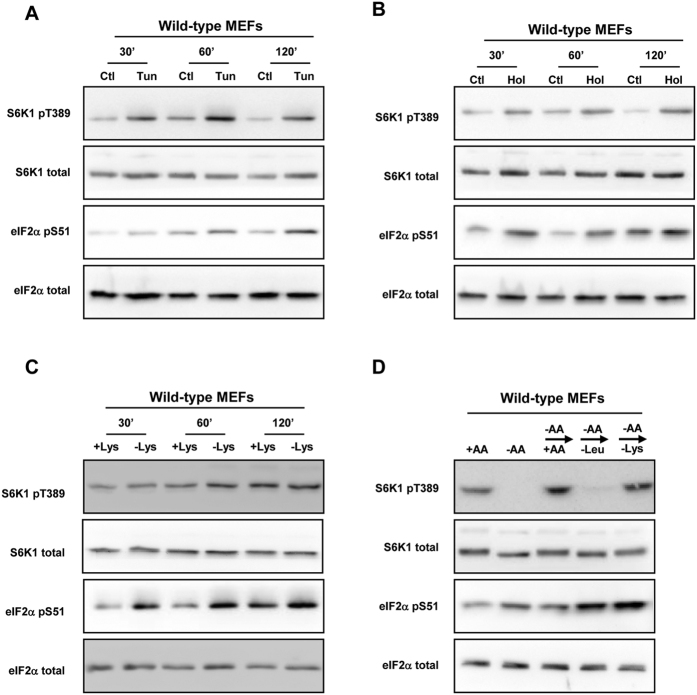
The phosphorylation of eIF2α is not sufficient to inhibit mTORC1. (**A**) WT MEFs were treated without (Ctl) or with tunicamycin (Tun) for the times shown. (**B**) WT MEFs were treated without (Ctl) or with histidinol (Hol) for the times shown. (**C**) WT MEFs were cultured in the presence or in absence of lysine (Lys) with all the other AAs for the indicated times. (**D**) WT MEFs were cultured in the presence or absence of AA for 75 min (+AA); or in absence of AA for 60 min prior to addition for 15 min of a medium containing all AA (−AA → + AA) or a medium containing all AA except leucine (−AA → -Leu) or lysine (−AA → -Lys). Immunoblot analyses were performed to measure the amounts of the indicated proteins and their levels of phosphorylation.

We subsequently investigated whether inducing the phosphorylation of eIF2α was sufficient to inhibit mTORC1. We used tunicamycin to increase eIF2α phosphorylation; this drug induces endoplasmic reticulum stress and the activation of a different eIF2α kinase, PERK. As expected, this treatment increased the phosphorylation of eIF2α, but also the phosphorylation of S6K1 (Fig. 4A). This could perhaps be explained by the fact that eIF2α phosphorylation inhibits translation and so can evoke a transient rise in the intracellular levels of amino acids that in turn induces the activation of mTORC1. This phenomenon has been already observed with another protein synthesis inhibitor, cycloheximide^22^. These results demonstrate that the induction of eIF2α phosphorylation by itself is not sufficient to inhibit mTORC1 activity.

The finding that the phosphorylation of eIF2α is necessary but not sufficient to inhibit mTORC1 led us to postulate that the activation of GCN2 alone might be not sufficient. To test this hypothesis, cells were treated with histidinol, a structural analogue of histidine that inhibits the charging of the histidyl-tRNA^23^ and in consequence activates GCN2. A induction of eIF2α phosphorylation was observed in cells treated with histidinol (Fig. 4B). This was accompanied by an induction of the phosphorylation of S6K1, which might again be explained by a transient rise in the intracellular level of amino acids due to inhibition of protein synthesis (and thus of amino acid utilization)^22^. As hypothesized, activation of GCN2 by itself does not seem sufficient to inhibit mTORC1 activity. In order to confirm this, cells were starved for lysine, since lysine deprivation efficiently induces the GCN2/ATF4 pathway in MEFs^24^ although there were no clear data concerning the effect of this condition on mTORC1 signaling. Indeed, despite the increase in eIF2α phosphorylation induced by lysine deprivation, we did not observe any change in S6K1 phosphorylation (Fig. 4C). Moreover, following the inhibition of S6K1 phosphorylation by amino acid deprivation, medium lacking lysine induced S6K1 phosphorylation to the same extent as the complete medium (Fig. 4D). These results confirm that the activation of GCN2 does not necessarily result in mTORC1 inhibition, even though both GCN2 and phosphorylation of eIF2α are required for inhibition of mTORC1. The corollary of this observation is that additional events controlled by leucine deprivation are required to inhibit mTORC1 activity.

### GCN2 is also necessary for the inhibition of mTORC1 activity during arginine deprivation

**Figure 5:**
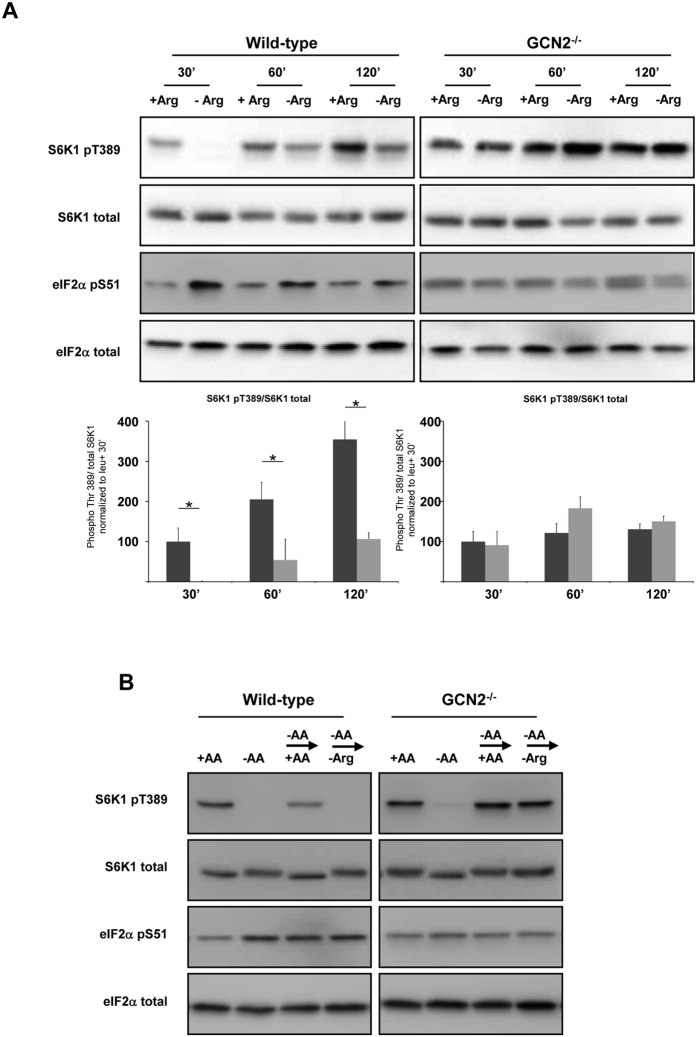
GCN2 is necessary for the regulation of mTORC1 by arginine. (**A**) WT or GCN2^−/−^ MEFs were cultured in presence or in absence of arginine (Arg) with all the other AAs for 30, 60 or 120 min. The ratio of phosphorylated S6K1 (Thr389) to total S6K1 was determined by densitometry analysis, differences between control cells (dark bars) and arginine starved cells (light bars) at each time point were assessed by 1-way ANOVA. Bars with (*) are significantly different from each other (*P* < 0.05). (**B**) WT MEFs and GCN2^−/−^ MEFs were cultured in the presence or absence of AA for 75 min; or in absence of AA for 60 min prior to addition for 15 min of a medium containing all AA (−AA → + AA) or a medium containing all AA except arginine (−AA → -Arg). Immunoblot analyses were performed to assess the amounts of the indicated proteins and their levels of phosphorylation.

We wondered whether the role of GCN2 was restricted only to leucine deprivation. Among amino acids, it is well known that arginine is also potent regulator of mTORC1 activity. Moreover, previous studies have demonstrated that arginine can control eIF2α phosphorylation^25^. We therefore tested the contribution of GCN2 to the regulation of mTORC1 by arginine. The data in Fig. 5A illustrate that, in WT MEFs, arginine deprivation induces eIF2α phosphorylation and decreases S6K1 phosphorylation. In contrast, in GCN2^−/−^ cells, the regulation of S6K1 phosphorylation by arginine was abolished. The same observation was made for 4E-BP1 and mTOR phosphorylation (Fig. S4). Then, the experimental model of starvation/stimulation was used. Following amino acid deprivation, the addition of a medium containing all amino acids except arginine was unable to induce S6K1 phosphorylation in WT MEFs, whereas this same medium efficiently induced the phosphorylation of S6K1 in GCN2^−/−^ MEFs (Fig. 5B). All these data demonstrate that GCN2 is also necessary for the regulation of mTORC1 by arginine and thus that the role of GCN2 in the control of mTORC1 is not restricted to leucine deprivation.

## Discussion

Our study reinforces the conclusion that regulation of mTORC1 by amino acids is complicated and is not limited to a single mechanism^26^. This is underscored by the fact that the regulation of mTORC1 by all amino acids differs from its control by individual amino acids such as leucine. The present study demonstrates that GCN2 plays an indispensable role in the inhibition of mTORC1 activity in response to leucine or arginine deprivation in mouse embryonic fibroblasts. However, our study also shows that activation of GCN2 is not itself sufficient to inhibit mTORC1. The observation that lysine deprivation over a short time period did not inhibit mTORC1 whereas it did induce GCN2 activation clearly illustrates this. It remains puzzling, in terms of biological significance, that deprivation for some amino acids induces GCN2 activation and inhibits mTORC1 whereas starvation for others only activates GCN2. It is very important to bear in mind that the ability of a given amino acid to control mTORC1 may differ according to the cell type. This illustrates the challenges of understanding the regulation of mTORC1 by single amino acids. Understanding this evident complexity is made harder by the fact that various different protocols have been used to study this regulation, e.g., cells maintained in the presence or absence of serum, or studying the effect of an amino acid in the presence or in the absence of other amino acids. These aspects might well be confounding when the results of different studies are evaluated.

One major finding of our study is that GCN2 acts in the early stages of mTORC1 inhibition independently of ATF4 or ongoing transcription. Thus, while one might speculate that transcriptional targets of ATF4 such REDD1^18^ could be involved in the inhibition of mTORC1 under such conditions, our results clearly demonstrate that GCN2 contributes to the early stages of mTORC1 inhibition in the absence of ATF4 and even of ongoing transcriptional activity. Importantly, our data do not contradict the possibility that the ATF4 target genes may contribute to maintaining mTORC1 inhibition upon prolonged leucine deprivation. Indeed, a faster recovery of S6K1 phosphorylation seems to occur in ATF4^−/−^ MEFs after 2 h of leucine deprivation (Fig. 2A). In the context of serum deprivation, it has been demonstrated that REDD1 sustains the repression of mTORC1 but is not responsible for the initial inhibition^18^.

According to our results obtained with eIF2α^S51A/S51A^ MEFs, the phosphorylation of eIF2α is necessary for the inhibition of mTORC1 signaling in amino acid-deprived cells. However, given that lack of need for ATF4, which can be translationally upregulated following eIF2α phosphorylation, the mechanism by which phosphorylation of eIF2α controls mTORC1 remains to be identified. The phosphorylation of eIF2α inhibits the guanine nucleotide-exchange activity of eIF2B; consequently, eIF2 remains in its inactive GDP-bound form and the formation of the 43S ribosomal preinitiation complex is abolished^27^. Holtz *et al.*^28^ have described that mTOR interacts with a multisubunit translation factor, eIF3, to regulate the formation of the preinitiation complex. Thus, the inhibition of the formation of the 43S preinitiation complex by eIF2α phosphorylation could impact on the localization of mTORC1 and thus on its regulation. Interestingly, a study which was published while we were finalizing our manuscript^29^, emphasizes the importance of the translation pre-initiation complex, in particularly eIF4A, in the regulation of mTORC1 activity.

The fact that GCN2 activation is necessary but not sufficient to inhibit mTORC1 signaling indicates the existence of additional mechanisms invoked by leucine or arginine deprivation^30^,^31^. Concerning arginine, the recent study by Wang *et al.*^30^ demonstrated that the amino acid transporter SCL38A9 is responsible for the regulation of mTORC1 specifically by arginine. This transporter is located at the lysosomal membrane and interacts with the Ragulator complex and the Rag proteins. Several mechanisms have been proposed to explain the regulation of mTORC1 by leucine, all of which are thought to rely upon the regulation of the Rag proteins and the localization of mTORC1 at the lysosome^32^,^33^. Finally, while we were finalizing our study, Wolfson *et al.*^34^ established that Sestrin2 (*sesn2*), an inhibitor of mTORC1 activity, is a sensor of leucine availability and that its presence is necessary for mTORC1 regulation by leucine availability. Even more recently, Ye *et al.*^35^ demonstrated that *sesn2* is a target gene of the GCN2/ATF4 pathway and that its transcriptional activation is required to sustain mTORC1 inhibition upon leucine deprivation. The authors also showed that sesn2 is needed for signaling the inhibition of mTORC1 upon long-term deprivation (24 h) of other amino acids such arginine. Given that Wolfson *et al.*^34^ demonstrated that Sestrin2 does not bind arginine, it will be important to determine how the increase of the expression of Sestrin2 contributes to the inhibitory effect of 24 h arginine deprivation.

It remains difficult to establish a unifying model that integrates the available data concerning the roles of GCN2 and Sestrin2 in the regulation of mTORC1 by individual amino acids. Concerning the setting of leucine deprivation, Sestrin2 appears to be a crucial regulator of mTORC1 activity through its ability to bind leucine^34^. In addition, it is clear that GCN2 and ATF4 contribute to the regulation of mTORC1 by Sestrin2 through the induction of its expression during leucine deprivation^35^. Nevertheless, as discussed above, GCN2 provides an additional input to mTORC1 signaling that does not require ATF4 expression or, therefore, the induction of *sesn2*. This interpretation is reinforced by the fact that GCN2 is also necessary for inhibition of mTORC1 upon short-term arginine deprivation whereas Sestrin2 does not appear to be directly involved in sensing arginine or the effects of arginine on mTORC1 signaling^34^.

The present study establishes that, in the context of mTORC1 regulation, GCN2 is a crucial sensor, probably of the cytoplasmic levels of leucine and arginine, which, among the amino acids, are the most potent regulators of mTORC1^36^. Our results complement the recent advances in the understanding of the mechanisms involved in sensing these two amino acids. However, it remains unresolved how GCN2, independently of ATF4, cooperates with sensing systems involving Sestrin2 and SLC38A9. It should be borne in mind that the relative contributions of these inputs may differ according the cell type and/or the model of amino acid deprivation/stimulation. It will be important to determine if the role of GCN2 during leucine or arginine deprivation is related to the control of mTORC1 at the level of lysosome, since the ability of mTOR to interact with the lysosome also appears to be crucial for mTORC1 inhibition by amino acid starvation^12^,^37^. Indeed, we previously observed a strong attenuation of the inhibitory effect of leucine deprivation on mTORC1 signaling in cells knocked down for RagC/D^12^. In addition, Ye *et al.*^35^ observed that leucine deprivation was less efficient in decreasing S6K1 phosphorylation in RagA/B-KO cells. A corollary of these experiments is the likely existence of Rag-independent inputs to the control of mTORC1. It is conceivable that GCN2 activation contributes to the regulation of mTORC1 in another cellular compartment such the Golgi apparatus^13^.

Our findings add significantly to the body of information on the control of mTORC1 by showing that GCN2 and the phosphorylation of its substrate eIF2α are important for the control of mTORC1 by leucine, but that this can occurs independently of the best-known target of the GCN2/eIF2 pathway, the transcription factor ATF4.

## Materials and Methods

### Reagents

Antibodies against S6K1 (#9202), S6K phosphorylated at Thr-389 (#9205), eIF2α (#9722) were from Cell Signaling Technology, antibody against eIF2α phosphorylated at Ser-51 (ab32157) was from Abcam. Dulbecco’s Modified Eagle’s Medium (DMEM) and Dulbecco’s Modified Eagle’s Medium/Nutrient F-12 Ham’s DMEM/F12 used for leucine deprived medium were from Sigma. DMEM without amino acids was from Genaxxon. DMEM used for arginine deprived medium was from ThermoFisher Scientific. Fetal bovine serum (FBS) was from Gibco and had been dialysed (using a membrane with a cut-off of 3500 Da) against phosphate-buffered saline (PBS) (pH: 7.4) at 4 °C. MEFs deficient in GCN2^21^ and ATF4^38^ were kindly donated by Prof. D. Ron (Institute of Metabolic Science, Cambridge, UK), eIF2α^S51A/S51A ^39 MEFs were kindly provided by Dr R. J. Kaufman (La Jolla, California, University of Michigan, USA). Actinomycin D, tunicamycin and histidinol were from Sigma.

### Cell culture and treatments

Mouse embryonic fibroblasts (MEFs) were grown in high glucose (4.5 g/liter) DMEM with 10% (v/v) FBS, 2 mM L-glutamine, mixture of MEM Non-essential Amino Acids (Invitrogen), 100 units/ml streptomycin sulfate, and 100 units/ml penicillin G, at 37 °C with 5% (v/v) CO_2._ For amino acid starvation experiments, cells were grown in DMEM containing high glucose and lacking all amino acids in the presence or absence of 10% (v/v) dialysed FBS. Cells were washed twice with PBS before starvation. The amino acid stimulation was performed by changing the ‘starved’ medium for complete DMEM medium in the presence or absence of 10% dialysed FBS. For leucine starvation, cells were grown in DMEM/F12 lacking leucine in the presence or absence of 10% dialysed FBS. Cells were rinsed twice with PBS before starvation. Leucine stimulation was performed by adding leucine at a final concentration of 450 μM to the leucine-free medium. All the experiments were repeated at least three times, and those using siRNA were repeated at least five times.

### Protein analysis

For protein extraction, lysis was performed with our standard lysis buffer (50 mM Tris, 25 mM β-glycerophosphate, 50 mM NaCl, 1 mM EDTA, 1 mM EGTA, 0.5% (v/v) Triton X100, 1 mM DTT, 1 mM benzamidine, protease and phosphatase inhibitor cocktail (Sigma). SDS-PAGE was performed as previously described^12^. The total protein concentrations were determined using a standard Bradford protein assay. Proteins were transferred onto PVDF membrane which was then blocked in Tris-Buffered Saline (TBS) containing 0.1% Tween20 and 5% (w/v) non-fat dry milk powder for 1 h at room temperature. The membrane was incubated with primary antibody overnight at 4 °C in TBS; 0.1% Tween20, 5% (w/v) non-fat dry milk or 5% (w/v) bovine serum albumin (BSA). Antibodies were diluted according to the manufacturer’s instructions. Membranes were washed 4 times for 5 min in TBS, 0.1% Tween20. Secondary antibodies coupled to HRP, diluted at 1/5000 in TBS, 0.1% Tween20, 5% (w/v) non-fat dried milk, was incubated at room temperature for 1 h. Membranes were washed 4 times for 5 min in TBS, 0.1% Tween20. A final wash of 5 min was performed in TBS. Luminata western HRP substrate (Millipore) and a chemiluminescence imager (G:box, Syngene) were used to detect the signals.

### Statistical analyses

All data are expressed as means ± SEM. Differences between sample of a same time of treatment and same genotype were assessed by one-way ANOVA (fisher test). Significance was defined at the 0.05 level.

## Additional Information

**How to cite this article**: Averous, J. *et al.* GCN2 contributes to mTORC1 inhibition by leucine deprivation through an ATF4 independent mechanism. *Sci. Rep.* **6**, 27698; doi: 10.1038/srep27698 (2016).

## Supplementary Material

Supplementary Information

## References

[b1] Kim D.-H. *et al.* mTOR interacts with raptor to form a nutrient-sensitive complex that signals to the cell growth machinery. Cell 110, 163–175 (2002). 12150925 10.1016/s0092-8674(02)00808-5

[b2] Burnett P. E., Barrow R. K., Cohen N. A., Snyder S. H. & Sabatini D. M. RAFT1 phosphorylation of the translational regulators p70 S6 kinase and 4E-BP1. Proc. Natl. Acad. Sci. USA 95, 1432–1437 (1998). 9465032 10.1073/pnas.95.4.1432 PMC19032

[b3] Hara K. *et al.* Amino Acid Sufficiency and mTOR Regulate p70 S6 Kinase and eIF-4E BP1 through a Common Effector Mechanism. J. Biol. Chem. 273, 14484–14494 (1998). 9603962 10.1074/jbc.273.23.14484

[b4] Berlanga J. J., Santoyo J. & de Haro C. Characterization of a mammalian homolog of the GCN2 eukaryotic initiation factor 2alpha kinase. Eur. J. Biochem. 265, 754–762 (1999). 10504407 10.1046/j.1432-1327.1999.00780.x

[b5] Vattem K. M. & Wek R. C. Reinitiation involving upstream ORFs regulates ATF4 mRNA translation in mammalian cells. Proc Natl Acad Sci USA 101, 11269–74 (2004). 15277680 10.1073/pnas.0400541101 PMC509193

[b6] Lopez A. B. *et al.* A feedback transcriptional mechanism controls the level of the arginine/lysine transporter cat-1 during amino acid starvation. Biochem. J. 402, 163–173 (2007). 17042743 10.1042/BJ20060941 PMC1783987

[b7] Siu F., Bain P. J., LeBlanc-Chaffin R., Chen H. & Kilberg M. S. ATF4 is a mediator of the nutrient-sensing response pathway that activates the human asparagine synthetase gene. J Biol Chem 277, 24120–7 (2002). 11960987 10.1074/jbc.M201959200

[b8] B’Chir W. *et al.* The eIF2alpha/ATF4 pathway is essential for stress-induced autophagy gene expression. Nucleic Acids Res 41, 7683–99 (2013). 23804767 10.1093/nar/gkt563 PMC3763548

[b9] Ham D. J., Lynch G. S. & Koopman R. Amino acid sensing and activation of mechanistic target of rapamycin complex 1: implications for skeletal muscle. Curr. Opin. Clin. Nutr. Metab. Care 19, 67–73 (2016). 26560525 10.1097/MCO.0000000000000240

[b10] Sancak Y. *et al.* The Rag GTPases Bind Raptor and Mediate Amino Acid Signaling to mTORC1. Science 320, 1496–1501 (2008). 18497260 10.1126/science.1157535 PMC2475333

[b11] Sancak Y. *et al.* Ragulator-Rag Complex Targets mTORC1 to the Lysosomal Surface and is Necessary for its Activation by Amino Acids. Cell 141, 290–303 (2010). 20381137 10.1016/j.cell.2010.02.024 PMC3024592

[b12] Averous J. *et al.* Requirement for lysosomal localization of mTOR for its activation differs between leucine and other amino acids. Cell. Signal. 26, 1918–1927 (2014). 24793303 10.1016/j.cellsig.2014.04.019

[b13] Thomas J. D. *et al.* Rab1A is an mTORC1 Activator and a Colorectal Oncogene. Cancer Cell 26, 754–769 (2014). 25446900 10.1016/j.ccell.2014.09.008 PMC4288827

[b14] Fox H. L., Kimball S. R., Jefferson L. S. & Lynch C. J. Amino acids stimulate phosphorylation of p70S6k and organization of rat adipocytes into multicellular clusters. Am J Physiol 274, C206–13 (1998). 9458729 10.1152/ajpcell.1998.274.1.C206

[b15] Dong J., Qiu H., Garcia-Barrio M., Anderson J. & Hinnebusch A. G. Uncharged tRNA activates GCN2 by displacing the protein kinase moiety from a bipartite tRNA-binding domain. Mol Cell 6, 269–79 (2000). 10983975 10.1016/s1097-2765(00)00028-9

[b16] Xiao F. *et al.* Leucine Deprivation Increases Hepatic Insulin Sensitivity via GCN2/mTOR/S6K1 and AMPK Pathways. Diabetes 60, 746–756 (2011). 21282364 10.2337/db10-1246 PMC3046835

[b17] Anthony T. G. *et al.* Preservation of Liver Protein Synthesis during Dietary Leucine Deprivation Occurs at the Expense of Skeletal Muscle Mass in Mice Deleted for eIF2 Kinase GCN2. J. Biol. Chem. 279, 36553–36561 (2004). 15213227 10.1074/jbc.M404559200

[b18] Dennis M. D., McGhee N. K., Jefferson L. S. & Kimball S. R. Regulated in DNA damage and development 1 (REDD1) promotes cell survival during serum deprivation by sustaining repression of signaling through the mechanistic target of rapamycin in complex 1 (mTORC1). Cell. Signal 25, 2709–2716 (2013). 24018049 10.1016/j.cellsig.2013.08.038 PMC3867791

[b19] Minami K. *et al.* Suppression of viral replication by stress-inducible GADD34 protein via the mammalian serine/threonine protein kinase mTOR pathway. J. Virol. 81, 11106–11115 (2007). 17670836 10.1128/JVI.01063-07 PMC2045534

[b20] Mordier S., Deval C., Bechet D., Tassa A. & Ferrara M. Leucine limitation induces autophagy and activation of lysosome-dependent proteolysis in C2C12 myotubes through a mammalian target of rapamycin-independent signaling pathway. J Biol Chem 275, 29900–6 (2000). 10893413 10.1074/jbc.M003633200

[b21] Harding H. P. *et al.* Regulated translation initiation controls stress-induced gene expression in mammalian cells. Mol Cell 6, 1099–108. (2000). 11106749 10.1016/s1097-2765(00)00108-8

[b22] Beugnet A., Tee A. R., Taylor P. M. & Proud C. G. Regulation of targets of mTOR (mammalian target of rapamycin) signalling by intracellular amino acid availability. Biochem. J. 372, 555–566 (2003). 12611592 10.1042/BJ20021266 PMC1223408

[b23] Hansen B. S., Vaughan M. H. & Wang L. Reversible inhibition by histidinol of protein synthesis in human cells at the activation of histidine. J. Biol. Chem. 247, 3854–3857 (1972). 4338230

[b24] Chaveroux C. *et al.* Identification of a novel amino acid response pathway triggering ATF2 phosphorylation in mammals. Mol Cell Biol 29, 6515–26 (2009). 19822663 10.1128/MCB.00489-09 PMC2786873

[b25] Lee J., Ryu H., Ferrante R. J., Morris S. M. & Ratan R. R. Translational control of inducible nitric oxide synthase expression by arginine can explain the arginine paradox. Proc. Natl. Acad. Sci. 100, 4843–4848 (2003). 12655043 10.1073/pnas.0735876100 PMC153643

[b26] Shimobayashi M. & Hall M. N. Multiple amino acid sensing inputs to mTORC1. Cell Res. 26, 7–20 (2016). 26658722 10.1038/cr.2015.146 PMC4816134

[b27] Sokabe M. & Fraser C. S. Human Eukaryotic Initiation Factor 2 (eIF2)-GTP-Met-tRNAi Ternary Complex and eIF3 Stabilize the 43 S Preinitiation Complex. J. Biol. Chem. 289, 31827–31836 (2014). 25246524 10.1074/jbc.M114.602870 PMC4231660

[b28] Holz M. K., Ballif B. A., Gygi S. P. & Blenis J. mTOR and S6K1 mediate assembly of the translation preinitiation complex through dynamic protein interchange and ordered phosphorylation events. Cell 123, 569–580 (2005). 16286006 10.1016/j.cell.2005.10.024

[b29] Tsokanos F.-F. *et al.* eIF4A inactivates TORC1 in response to amino acid starvation. EMBO J. n/a–n/a. doi: 10.15252/embj.201593118 (2016). PMC4868951 26988032

[b30] Wang S. *et al.* Lysosomal amino acid transporter SLC38A9 signals arginine sufficiency to mTORC1. Science 347, 188–194 (2015). 25567906 10.1126/science.1257132 PMC4295826

[b31] Rebsamen M. *et al.* SLC38A9 is a component of the lysosomal amino acid-sensing machinery that controls mTORC1. Nature 519, 477–481 (2015). 25561175 10.1038/nature14107 PMC4376665

[b32] Han J. M. *et al.* Leucyl-tRNA Synthetase is an Intracellular Leucine Sensor for the mTORC1-Signaling Pathway. Cell 149, 410–424 (2012). 22424946 10.1016/j.cell.2012.02.044

[b33] Durán R. V. *et al.* Glutaminolysis Activates Rag-mTORC1 Signaling. Mol. Cell 47, 349–358 (2012). 22749528 10.1016/j.molcel.2012.05.043

[b34] Wolfson R. L. *et al.* Sestrin2 is a leucine sensor for the mTORC1 pathway. Science 351, 43–48 (2016). 26449471 10.1126/science.aab2674 PMC4698017

[b35] Ye J. *et al.* GCN2 sustains mTORC1 suppression upon amino acid deprivation by inducing Sestrin2. Genes Dev. 29, 2331–2336 (2015). 26543160 10.1101/gad.269324.115 PMC4691887

[b36] Hara K. *et al.* Amino acid sufficiency and mTOR regulate p70 S6 kinase and eIF-4E BP1 through a common effector mechanism. J Biol Chem 273, 14484–94 (1998). 9603962 10.1074/jbc.273.23.14484

[b37] Demetriades C., Doumpas N. & Teleman A. A. Regulation of TORC1 in Response to Amino Acid Starvation via Lysosomal Recruitment of TSC2. Cell 156, 786–799 (2014). 24529380 10.1016/j.cell.2014.01.024 PMC4346203

[b38] Harding H. P. *et al.* An integrated stress response regulates amino acid metabolism and resistance to oxidative stress. Mol. Cell 11, 619–633 (2003). 12667446 10.1016/s1097-2765(03)00105-9

[b39] Scheuner D. *et al.* Translational Control is Required for the Unfolded Protein Response and *In Vivo* Glucose Homeostasis. Mol. Cell 7, 1165–1176 (2001). 11430820 10.1016/s1097-2765(01)00265-9

