# Supplementary material for: GCN2 contributes to mTORC1 inhibition by leucine deprivation through an ATF4 independent mechanism
Source: Sci Rep. 2016 Jun 14;6:27698. doi: 10.1038/srep27698 (PMC4906353; doi:10.1038/srep27698)
Supplement: Supplementary Information [file srep27698-s1.pdf]

GCN2 contributes to mTORC1 inhibition by leucine deprivation through an ATF4 independent mechanism.

Julien Averous[1,2*†], Sarah Lambert-Langlais[1,2*], Florent Mesclon[1,2], Valérie Carraro[1,2], Laurent Parry [1,2], Céline Jousse[1,2], Alain Bruhat[1,2], Anne-Catherine Maurin[1,2], Philippe Pierre[3], Christopher G Proud[4], Pierre Fafournoux[1,2†].

Supplementary Materials

Reagents

Antibodies against mTOR (#2983), mTOR phosphorylated at Serine 2448 (#2971), 4E-BP1 (#9452) and 4E-BP1 phosphorylated at threonine 36/45 (#9459) were from Cell Signaling Technology, antibody against GCN2 phosphorylated at serine 998 (ab75836) was from abcam.

Supplementary Figures

Fig S1

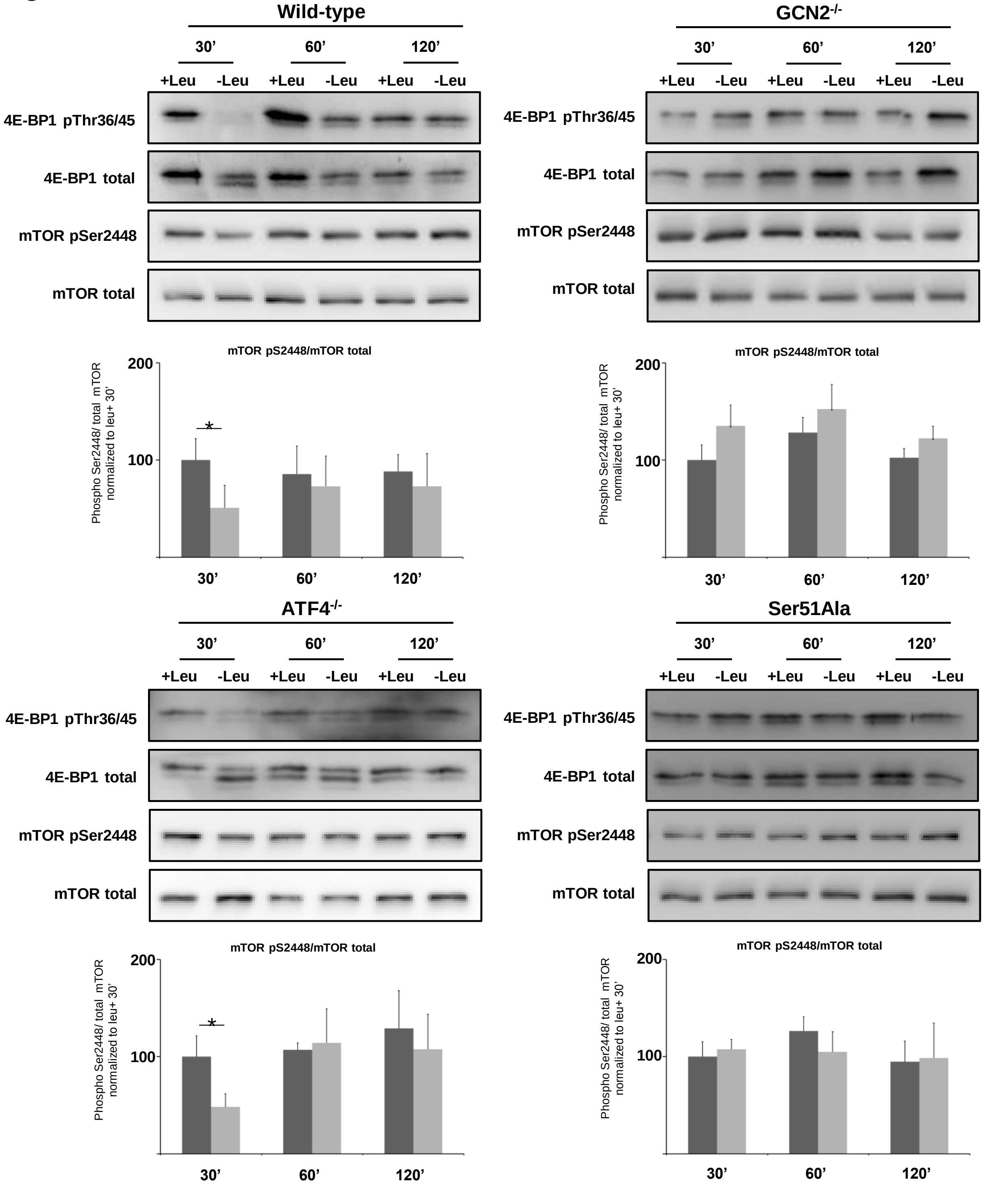

Figure S1 : Wild-Type (WT), GCN2-KO (-/-), ATF4-KO (-/-) or eIF2α [Ser51Ala] MEFs were cultured in presence or in absence of leucine (Leu) with all the other AAs for 30 min, 60 min or 120 min. Immunoblot analyses were performed on the resulting cell lysates using the indicated antibodies. The ratio of phosphorylated mTOR (Ser2448) to total mTOR was determined by densitometry analysis, differences between control cells and arginine starved cells at each time point were assessed by 1-way ANOVA. Bars with (*) are significantly different from each other ($P < 0.05$).

Fig S2

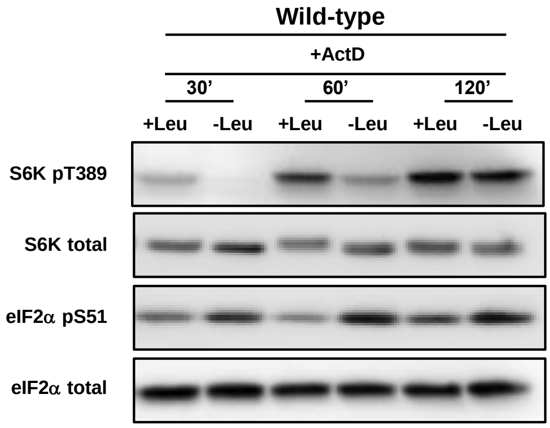

Figure S2: Wild-Type (WT) MEFs were cultured in presence or in absence of leucine (Leu) with all the other AAs for 30 min, 60 min or 120 min, with actinomycin D (1 µg/ml). . Immunoblot analyses were performed on the resulting cell lysates using the indicated antibodies.

Fig S3

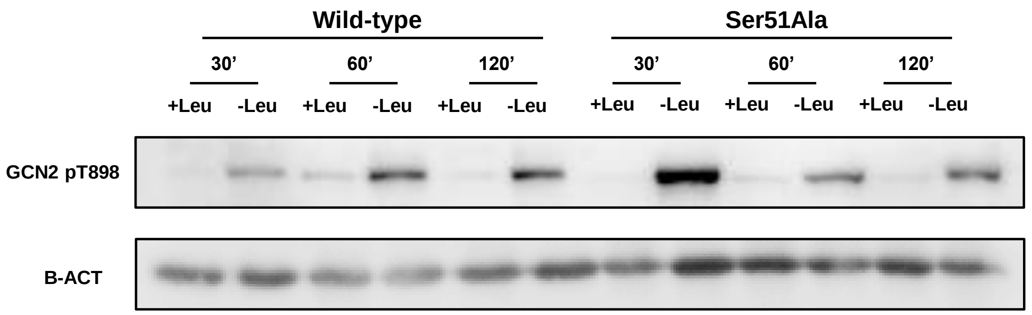

Figure S3 : Wild-Type (WT), or eIF2α [Ser51Ala] MEFs were cultured in presence or in absence of leucine (Leu) with all the other AAs for 30 min, 60 min or 120 min. Immunoblot analyses were performed on the resulting cell lysates using the indicated antibodies.

Fig S4

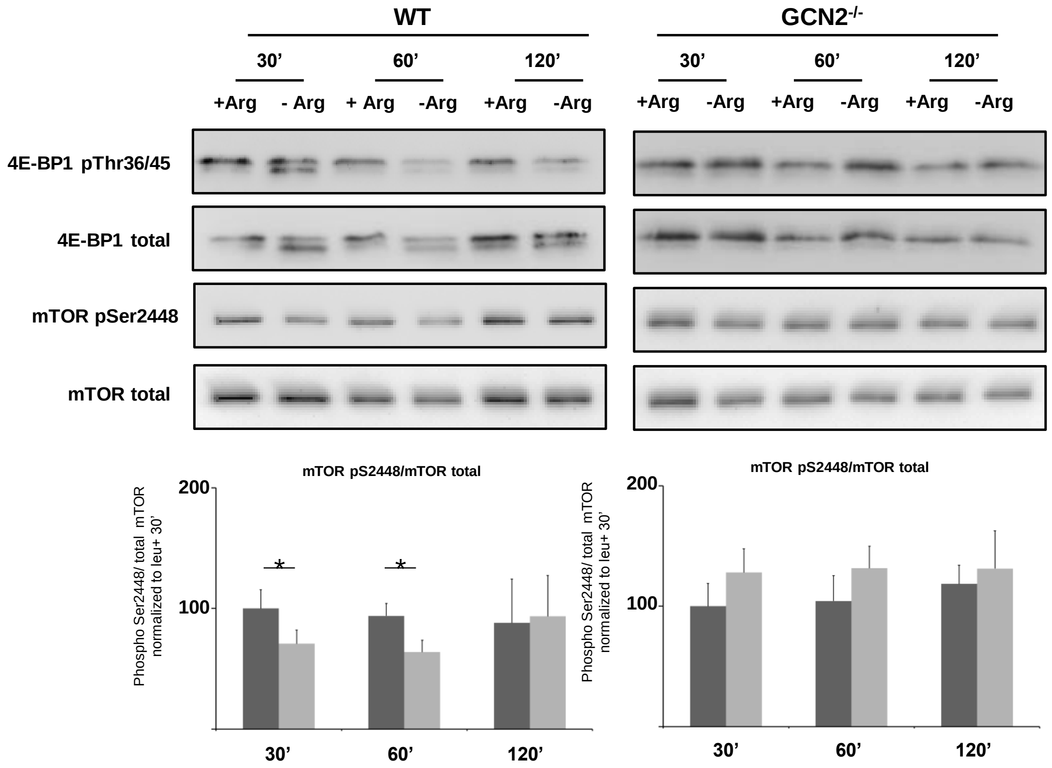

Figure S4 : Wilf Type (WT)or GCN2$^{-/-}$ MEFs were cultured in presence or in absence of arginine (Arg) with all the other AAs for 30 min, 60 min or 120 min. Immunoblot analyses were performed to assess the amounts of the indicated proteins and their levels of phosphorylation. The ratio of phosphorylated mTOR (Ser2448) to total mTOR was determined by densitometry analysis, differences between control cells and arginine starved cells at each time point were assessed by 1-way ANOVA. Bars with (*) are significantly different from each other (*P* < 0.05).
